# Ultrasound-based radiomics machine learning models for diagnosing cervical lymph node metastasis in patients with non-small cell lung cancer: a multicentre study

**DOI:** 10.1186/s12885-024-12306-6

**Published:** 2024-04-27

**Authors:** Zhiqiang Deng, Xiaoling Liu, Renmei Wu, Haoji Yan, Lingyun Gou, Wenlong Hu, Jiaxin Wan, Chenwanqiu Song, Jing Chen, Daiyuan Ma, Haining Zhou, Dong Tian

**Affiliations:** 1 Department of Thoracic Surgery, West China Hospital, Sichuan University, Chengdu, China; 2 Department of Oncology, Affiliated Hospital of North Sichuan Medical College, Nanchong, China; 3 College of Medical Imaging, North Sichuan Medical College, Nanchong, China; 4 Department of Ultrasound, Nanchong Central Hospital, Nanchong, China; 5 Department of Ultrasound, Suining Central Hospital, Suining, China; 6 Department of General Thoracic Surgery, Juntendo University School of Medicine, Tokyo, Japan; 7 Department of Ultrasound, Affiliated Hospital of North Sichuan Medical College, Nanchong, China; 8 Department of Clinical Medicine, North Sichuan Medical College, Nanchong, China; 9 Department of Thoracic Surgery, Suining Central Hospital, Sunning, China

**Keywords:** Non-small cell lung cancer, Ultrasound, Radiomics, Lymph node metastasis, Machine Learning

## Abstract

**Background:**

Cervical lymph node metastasis (LNM) is an important prognostic factor for patients with non-small cell lung cancer (NSCLC). We aimed to develop and validate machine learning models that use ultrasound radiomic and descriptive semantic features to diagnose cervical LNM in patients with NSCLC.

**Methods:**

This study included NSCLC patients who underwent neck ultrasound examination followed by cervical lymph node (LN) biopsy between January 2019 and January 2022 from three institutes. Radiomic features were extracted from the ultrasound images at the maximum cross-sectional areas of cervical LNs. Logistic regression (LR) and random forest (RF) models were developed. Model performance was assessed by the area under the curve (AUC) and accuracy, validated internally and externally by fivefold cross-validation and hold-out method, respectively.

**Results:**

In total, 313 patients with a median age of 64 years were included, and 276 (88.18%) had cervical LNM. Three descriptive semantic features, including long diameter, shape, and corticomedullary boundary, were selected by multivariate analysis. Out of the 474 identified radiomic features, 9 were determined to fit the LR model, while 15 fit the RF model. The average AUCs of the semantic and radiomics models were 0.876 (range: 0.781–0.961) and 0.883 (range: 0.798–0.966), respectively. However, the average AUC was higher for the semantic-radiomics combined LR model (0.901; range: 0.862–0.927). When the RF algorithm was applied, the average AUCs of the radiomics and semantic-radiomics combined models were improved to 0.908 (range: 0.837–0.966) and 0.922 (range: 0.872–0.982), respectively. The models tested by the hold-out method had similar results, with the semantic-radiomics combined RF model achieving the highest AUC value of 0.901 (95% CI, 0.886–0.968).

**Conclusions:**

The ultrasound radiomic models showed potential for accurately diagnosing cervical LNM in patients with NSCLC when integrated with descriptive semantic features. The RF model outperformed the conventional LR model in diagnosing cervical LNM in NSCLC patients.

**Supplementary Information:**

The online version contains supplementary material available at 10.1186/s12885-024-12306-6.

## Introduction

Lung cancer is the leading cause of cancer-related mortality worldwide; among its histologic subtypes, non-small cell lung cancer (NSCLC) is predominant [1, 2]. Although the widespread use of low-dose computed tomography for lung cancer screening is detecting early-stage NSCLC in more patients, more than half of patients are at an advanced stage when diagnosed [3–6]. In advanced NSCLC, the cervical lymph nodes (LNs) are common sites for distant metastasis [7]. Knowing the status of the cervical LNs is crucial for clinicians to make decisions for patients with NSCLC. According to the 8th TNM classification of lung cancer, NSCLC patients with metastasis in the lower and upper cervical LNs are categorized as stage IIIB and IV, respectively. The recommended first-line treatments for these patients are concurrent chemoradiotherapy, targeted therapy, or immunotherapy, while surgical intervention is inappropriate [8–10]. Therefore, identifying the status of the cervical LNs plays an integral role in accurate pretreatment staging and clinical decision-making for patients with NSCLC.

Neck ultrasound and ultrasound-guided biopsy are commonly used to identify the status of cervical LNs [11]. Although ultrasound is the preferred method for examining cervical lymph node metastasis (LNM), its diagnostic accuracy can be influenced by various factors. There is still room to improve the performance of ultrasound in diagnosing cervical LNM in NSCLC [12]. In patients with NSCLC, when suspected cervical LNM is detected by ultrasound, ultrasound-guided biopsy is recommended for further clinical investigation. Although ultrasound-guided biopsy is considered the gold standard for assessing the status of cervical LNs, it is limited by sampling errors and potential complications [11]. Therefore, a precise and noninvasive diagnostic approach is warranted to evaluate the status of cervical LNs.

Radiomics is a promising approach that utilizes quantitative features extracted from medical images to develop models aimed at supporting clinical decision-making [13]. Due to the high dimensionality of radiomic features, powerful analytical methods and tools are needed. As a vital branch of artificial intelligence, machine learning algorithms have the potential to enhance the performance of radiomics models [14]. However, there have been no studies applying ultrasound radiomics based on machine learning to the diagnosis of cervical LNM in NSCLC. We designed the present study to investigate the performance of models based on ultrasound radiomic features and/or descriptive semantic features in diagnosing cervical LNM in patients with NSCLC from three institutes. Additionally, we explored the ability of the machine learning algorithms to optimize model performance.

## Methods

### Patients

**Fig. 1:**
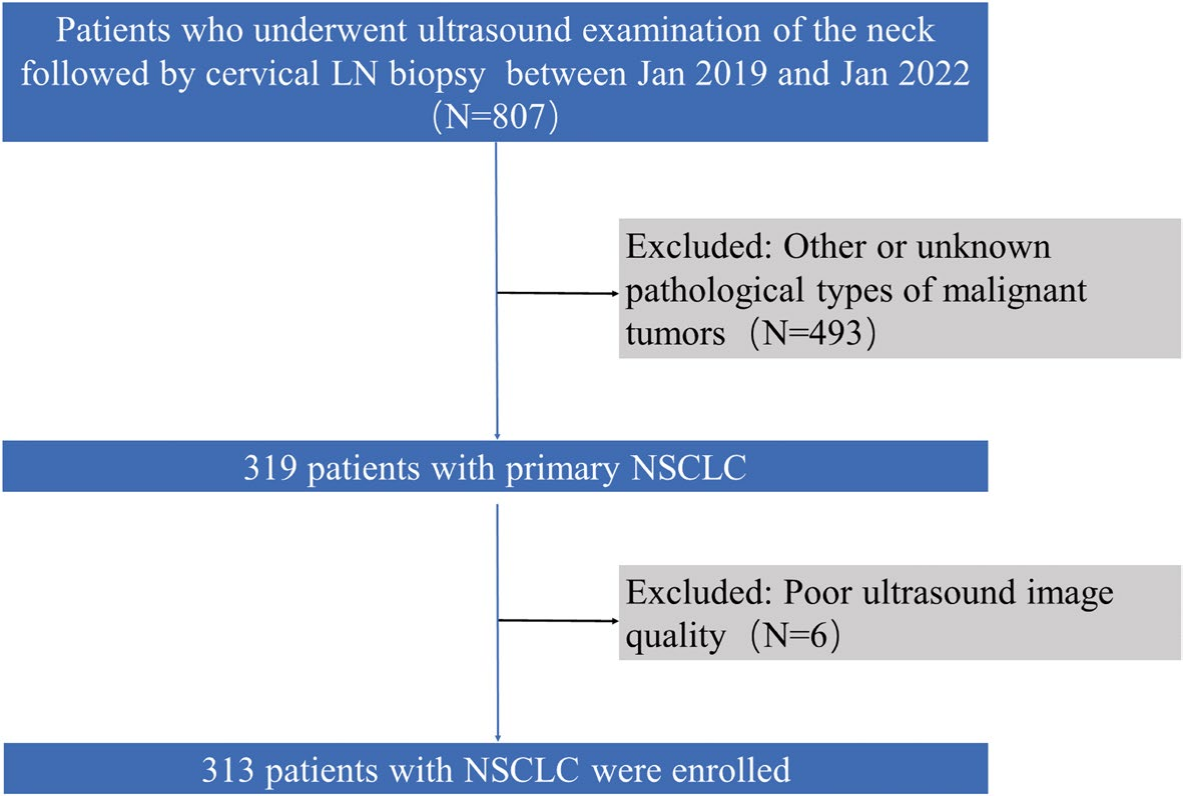
Flow chart of patient enrollment. LN, lymph node; NSCLC, non-small cell lung cancer

Patients with NSCLC who underwent neck ultrasound examination followed by cervical LN biopsy at Affiliated Hospital of North Sichuan Medical College, Nanchong Central Hospital, and Suining Central Hospital between January 2019 and January 2022 were initially recruited. The inclusion criteria were as follows: (1) cervical LN ultrasound examination followed by biopsy; (2) primary NSCLC; and (3) available detailed descriptive semantic characteristics. Patients with poor ultrasound image quality were excluded from the current study. A total of 807 patients who underwent neck ultrasound examination followed by cervical LN biopsy were initially included in the study, out of whom 319 with primary NSCLC were identified. After 6 patients were excluded due to poor-quality ultrasound images, the study finally enrolled 313 patients. The flow chart of participant inclusion and exclusion is shown in Fig. 1. The Ethics Committees and Review Board of the Nanchong Central Hospital, Affiliated Hospital of North Sichuan Medical College, and Suining Central Hospital approved this study. Patient consent was waived due to the retrospective nature of the study.

### Image acquisition and descriptive semantic characteristics collection

**Fig. 2:**
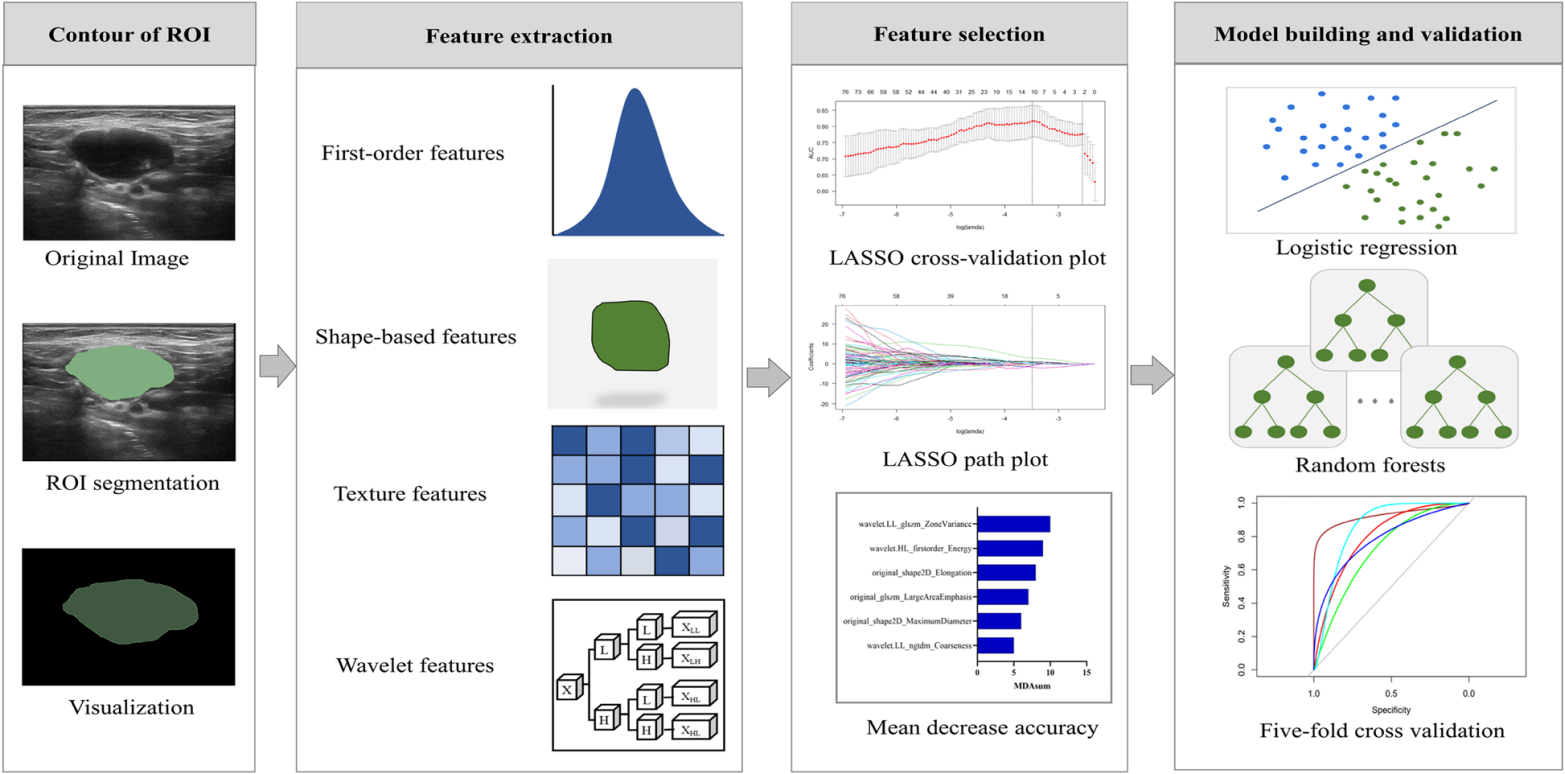
Workflow of the radiomics analysis. The LASSO was used to perform the feature selection of the logistic regression model, while the mean decrease accuracy for the random forests model

All enrolled patients underwent a routine pretreatment ultrasound examination with a Mindray Rezona 7 T (Shenzhen Mindray Bio-Medical Electronics Co, Ltd, China) or a GE Vivid E9 and E20 (General Electric Co, USA) ultrasound system. In addition, all ultrasound devices used a 6–15 L linear array probe with frequencies ranging from 5 to 12 MHz during the examination. We selected images containing the maximum LN cross-sections, which is considered the most useful radiologic criterion for assessing cervical LNM, for further radiomics analysis [15]. Descriptive semantic features were evaluated by two radiologists (X.L.L and R.M.W) and included nine parameters: the site (left and right), long diameter (largest diameter of the cervical LN), short diameter (vertical to the long diameter), shape (irregular and regular), boundary (unclear and clear), calcification (with calcification, without calcification), liquidation (with liquidation, without liquidation), corticomedullary boundary (unclear and clear), and lymph node location of the cervical LNs (according to the guidelines of the American Head and Neck Society and the American Academy of Otolaryngology in 2002) [16]. Demographic characteristics such as the sex and age of the patients were collected (Fig. 2).

### Segmentation and extraction of radiomic features

3D Slicer software version 4.10.2 (https://www.slicer.org) was used to segment ultrasound images manually. Regions of interest (ROIs) were independently segmented by two radiologists with 13 (X.L.L) and 10 (R.M.W.) years of experience in ultrasound. A final determination of the ROIs for each segmentation required the agreement of the two readers (X.L.L and R.M.W). Radiomic features were extracted from the ROIs on the ultrasound images using the PyRadiomics package version 1.2.0 (Computational Imaging and Bioinformatics Lab, Harvard Medical School) in Python version 3.7 (Python Software Foundation) [17]. A total of 474 radiomic features were extracted from each ultrasound image, including first-order, shape, textural, and wavelet features. The z score was determined d to normalize all radiomic features (Fig. 2).

### Feature selection and machine learning algorithm

Univariate and multivariate analyses were used to select descriptive semantic features and demographic characteristics. Univariate logistic regression (LR) was used to screen risk factors for cervical LNM, with features having a *P* < 0.10 included in the subsequent multivariate LR to identify independent risk factors (*P* < 0.05); odds ratios (OR) with 95% confidence intervals (Cl) were calculated. For radiomic features, a three-step feature selection method was employed. Unstable and low-reproducibility features were excluded by calculating the interclass correlation coefficient (ICC), and features with an ICC < 0.75 were removed. The nonparametric Mann‒Whitney U test was used to identify features significantly associated with cervical LNM (*P* < 0.05). Subsequently, the least absolute shrinkage and selection operator (LASSO) was used for the LR model to select the modeling features. The LASSO method decreases feature dimensionality while avoiding collinearity and overfitting [18]. We used fivefold cross-validation to determine the best-fitting λ, allowing the selection of features with nonzero coefficients for fitting the LR models. Feature selection for the random forests (RF) model was performed using the mean decrease accuracy (MDA), a measure for ranking feature importance in the RF algorithm. It directly measures the impact of each feature on the prediction or classification accuracy of the model; the higher the value, the more important the feature is [19]. We selected the 15 top-ranked features of the MDA sum for fitting the RF model. Model performance was assessed using ROC curve analysis, and the areas under the curve (AUCs) were calculated. Five-fold cross-validation was used to validate the model performance internally. In addition, the hold-out method was used to test the performance of models externally. It could evaluate the reproducibility of models and determine whether the models have similar performance in new data. The training set comprised patients from two centers, while patients from another were designated as the test set. All statistical analyses were performed using R (version 3.6.3, R Foundation for Statistical Computing). The AUCs and accuracies were described using two methods: the cross-validation method presents the average value with range, and the hold-out method presents a specific value along with its 95% CI. Quantitative, continuous, and categorical variables are expressed as medians and ranges, mean ± SD, and frequencies with proportions, respectively. A two-tailed *P* value < 0.05 indicated statistical significance. The workflow of the radiomics analysis is shown in Fig. 2.

## Results

### Patient characteristics

In total, 313 patients were included, with a median age of 64 (range, 29–87) years, and 216 (69.0%) were male. Of the 313 patients, 276 (88.18%) had cervical LNM identified by biopsy. The long diameter (mean ± SD, 22.9 ± 0.6 vs. 13.8 ± 0.9, *P* < 0.001), short diameter (mean ± SD, 14.5 ± 0.4 vs. 8.3 ± 1.5, *P* < 0.001), shape regularity (*P* < 0.001), and corticomedullary boundary clarity (*P* < 0.001) were significantly different between the cervical LNM-positive group and cervical LNM-negative group. No statistically significant differences in age, sex, calcification, liquidation, lymph node site, or location were noted between the groups (*P* > 0.05). The number of cervical LNs that metastasized to the left and right sides of the neck was approximately equal (48.9% vs. 51.1%, *P* = 0.370). The most common cervical sites of LNM were lower jugular nodes (IV, 50.4%), spinal accessory nodes and supraclavicular nodes (V, 38.0%). Other descriptive semantic features are summarized in Table 1.

**Table 1:** The clinical and descriptive semantic characteristics of patients with non-small cell lung cancer

Parameters	All patients	Cervical LNs status		*P*
	**(*N* = 313)**	**Positive (*n* = 276)**	**Negative (*n* = 37)**	
Age(y) (median) (rang)	64 (29–87)	64 (38–87)	66 (29–83)	0.932^a^
Sex				0.091^a^
Male	216 (69.0%)	186 (67.4%)	30 (81.8%)	
Female	97 (31.0%)	90 (32.6%)	7 (18.9%)	
LD of cervical LNs(mm)	21.8 ± 0.6	22.9 ± 0.6	13.8 ± 0.9	< 0.001^b*^
SD of cervical LNs(mm)	13.8 ± 0.4	14.5 ± 0.4	8.3 ± 1.5	< 0.001^b*^
Shape				< 0.001^a*^
Irregular	190 (60.7%)	178 (64.5%)	12 (32.4%)	
Regular	123 (39.3%)	98 (35.5%)	25 (67.6%)	
Corticomedullary boundary				< 0.001^a*^
Unclear	307 (98.1%)	274 (99.3%)	33 (89.2%)	
Clear	6 (1.9%)	2 (0.7%)	4 (10.8%)	
Boundary				0.237^a^
Unclear	103 (32.9%)	94 (34.1%)	9 (24.3%)	
Clear	210 (67.1%)	182 (65.9%)	28 (75.7%)	
Calcification				0.405^a^
Negative	245 (78.3%)	218 (79.0%)	27 (73.0%)	
Positive	68 (21.7%)	58 (21.0%)	10 (27.0%)	
Liquidation				0.495^a^
Negative	282 (90.1%)	247 (89.5%)	35 (94.6%)	
Positive	31 (9.9%)	29 (10.5%)	2 (5.4%)	
Lymph node site				0.370^a^
Left	156 (49.8%)	135 (48.9%)	21 (56.8%)	
Right	157 (50.2%)	141 (51.1%)	16 (43.2%)	
Lymph node location^c^				0.054^a^
I	3 (1.0%)	3 (1.1%)	0 (0.0%)	
II	11 (3.5%)	9 (3.3%)	2 (5.4%)	
III	23 (7.3%)	19 (6.9%)	4 (10.8%)	
IV	157 (50.2%)	139 (50.4%)	18 (48.7%)	
V	116 (37.1%)	105 (38.0%)	11 (29.7%)	
VI	1 (0.3%)	0 (0.0%)	1 (2.7%)	
VII	2 (0.6%)	1 (0.3%)	1 (2.7%)	

Data are expressed as *n* (%) unless otherwise specified

*LNs* lymph nodes, *LD* long diameter, *SD* short diameter, *I* submental nodes and submandibular nodes, *II* upper jugular nodes, *III* middle jugular nodes, *IV* lower jugular nodes, *V* spinal accessory nodes and supraclavicular nodes, *VI* central lymph nodes, *VII* superior mediastinum lymph nodes

^*^P < 0.05

^a^Chi-square test

^b^Student’s test

^c^Lymph node location

### Feature selection

**Fig. 3:**
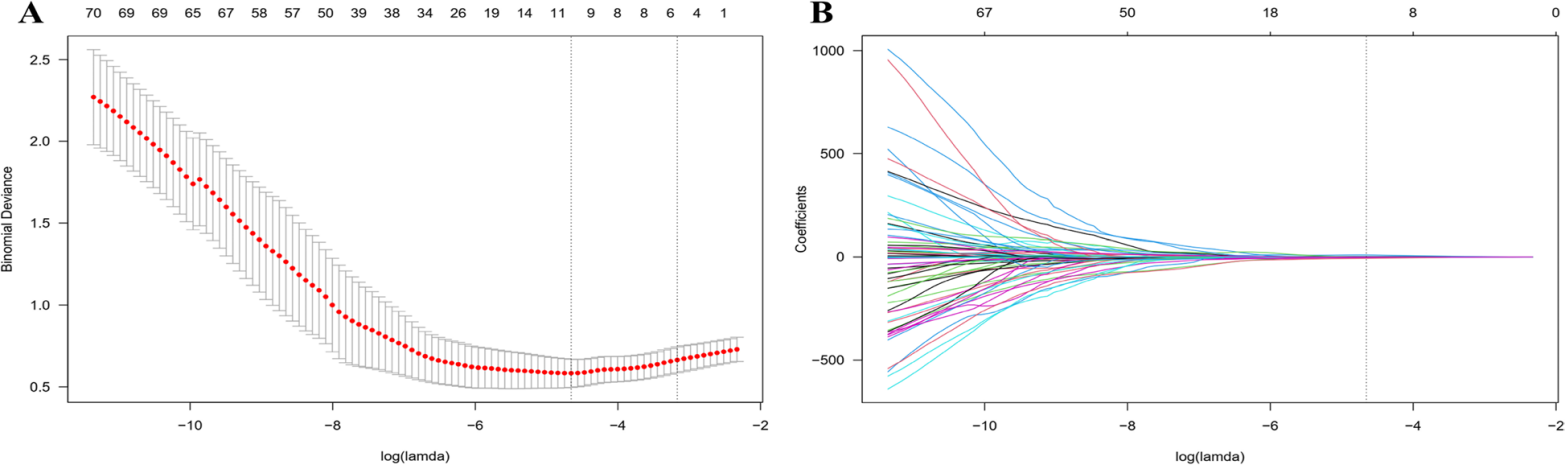
Figures of logistic least absolute shrinkage and selection operator (LASSO) regression. **A** Cross-validation plot for the penalty term. **B** LASSO path plot of the model in the dataset

**Fig. 4:**
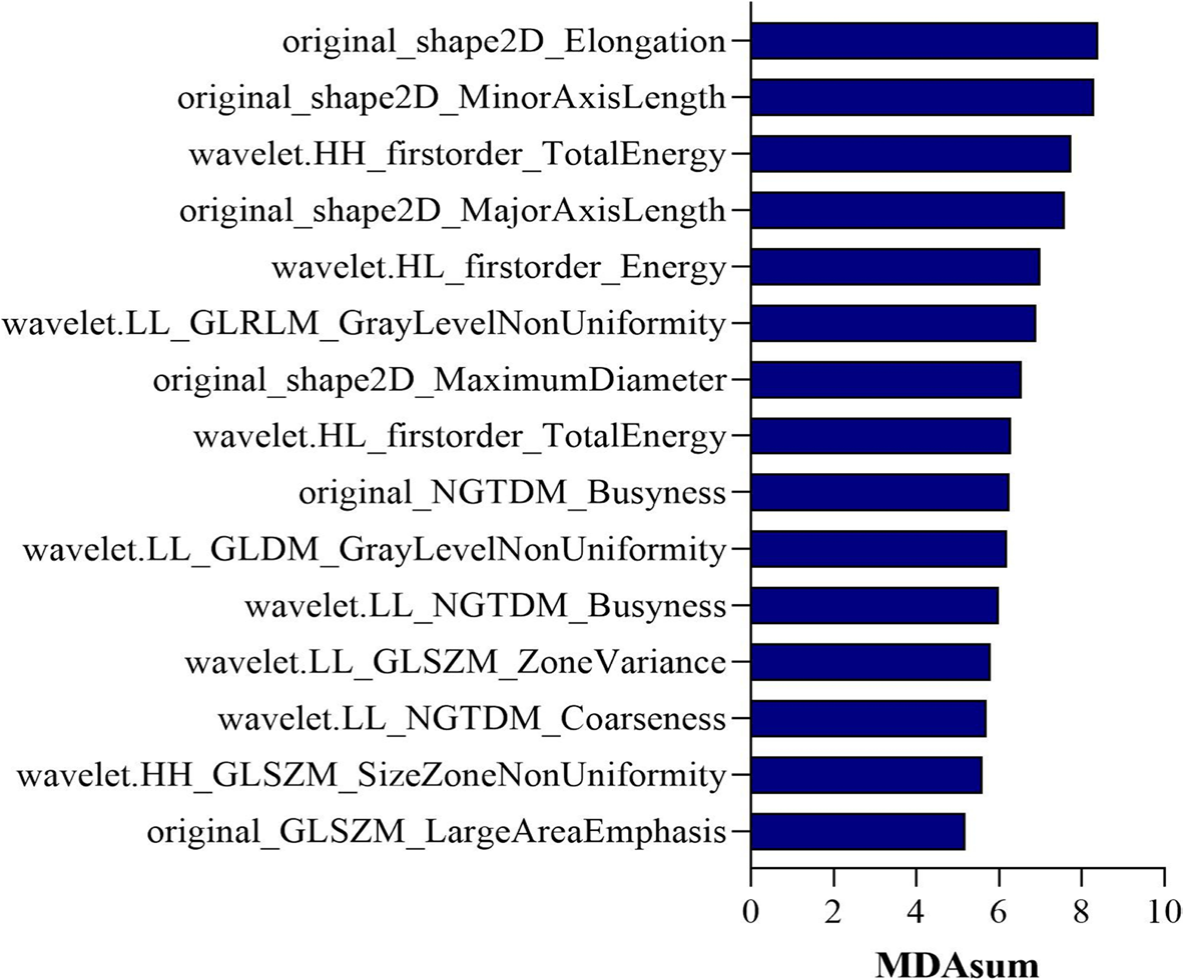
The mean decrease accuracy (MDA) of the 15 top-ranked features. Each of the 15 top-ranked features in the MDA sum was included in the final models for predicting cervical lymph node metastasis

The results of univariate and multivariate analyses are displayed in Table 2. Univariate analysis showed that one demographic feature and four descriptive semantic features, namely sex, long diameter, short diameter, shape, and corticomedullary boundary, had a *P* value < 0.10. After the multivariate analysis, three statistically significant factors were selected: long diameter (OR = 1.210, 95% Cl, 1.081–1.354, *P* = 0.001), shape (OR = 2.709, 95% Cl, 1.176–6.238, *P* = 0.019), and corticomedullary boundary (OR = 9.478, 95% Cl, 1.371–65.529, *P* = 0.023). Following the ICC evaluation and Mann‒Whitney U test, 104 of 474 radiomic features were selected for subsequent analyses. After the above two steps, among the remaining 104 features, nine modeling radiomic features were selected by the LASSO algorithm for fitting the LR model (Fig. 3), including two shape features, one texture feature, and 6 wavelet features (Table 3). For fitting the RF model, the 15 top-ranked features of the MDA sum were selected, including four shape features, two texture features, and nine wavelet features (Fig. 4). Furthermore, the original_shape2D_Elongation was the most critical feature among those selected by both LASSO and MDA-top15.

**Table 2:** Univariate and multivariate analyses for clinical risk factors of cervical lymph node metastasis

**Parameters**	**Univariate**	***P***	**Multivariate**	***P***
	**OR (95%Cl)**		**OR (95%Cl)**	
Age (y)	1.001 (0.968–1.036)	0.932	-	-
Sex (male/female)	0.482 (0.204–1.140)	0.097	0.500 (0.195–1.283)	0.149
LD of cervical LNs (mm)	1.211 (1.124–1.306)	< 0.001^*^	1.210 (1.081–1.354)	0.001^*^
SD of cervical LNs (mm)	1.355 (1.203–1.525)	< 0.001^*^	1.046 (0.907–1.207)	0.535
Shape (irregular/regular)	3.784 (1.821–7.861)	< 0.001^*^	2.709 (1.176–6.238)	0.019^*^
Corticomedullary Boundary (unclear/clear)	16.606 (2.928–94.174)	0.002^*^	9.478 (1.371–65.529)	0.023^*^
Boundary (unclear/clear)	1.607 (0.728–3.545)	0.240	-	-
Calcification (positive/negative)	0.718 (0.329–1.569)	0.407	-	-
Liquidation (positive/negative)	2.055 (0.470–8.990)	0.339	-	-
Lymph node site (left/right)	0.729 (0.365–1.457)	0.372	-	-

*OR* odds ratio, *CI* confidence interval, *LD* long diameter, *LNs* lymph nodes, *SD* short diameter

^*^P < 0.05

**Table 3:** Radiomic features involved in the logistic regression model

No	Feature categories	Feature names
1	Shape	original_shape2D_Elongation
2		original_shape2D_Minor Axis Length
3	Texture	original_GLSZM_Zone Entropy
4	Wavelet	wavelet.LH_GLSZM_Small Area Emphasis
5		wavelet.HL_GLDM_Dependence Entropy
6		wavelet.HL_GLDM_Dependence Non-Uniformity Normalized
7		wavelet.LL_GLSZM_Size Zone Non-Uniformity
8		wavelet.LH_GLRLM_Short Run Low Gray Level Emphasis
9		wavelet.HH_NGTDM_Contrast

### Model development and validation

**Fig. 5:**
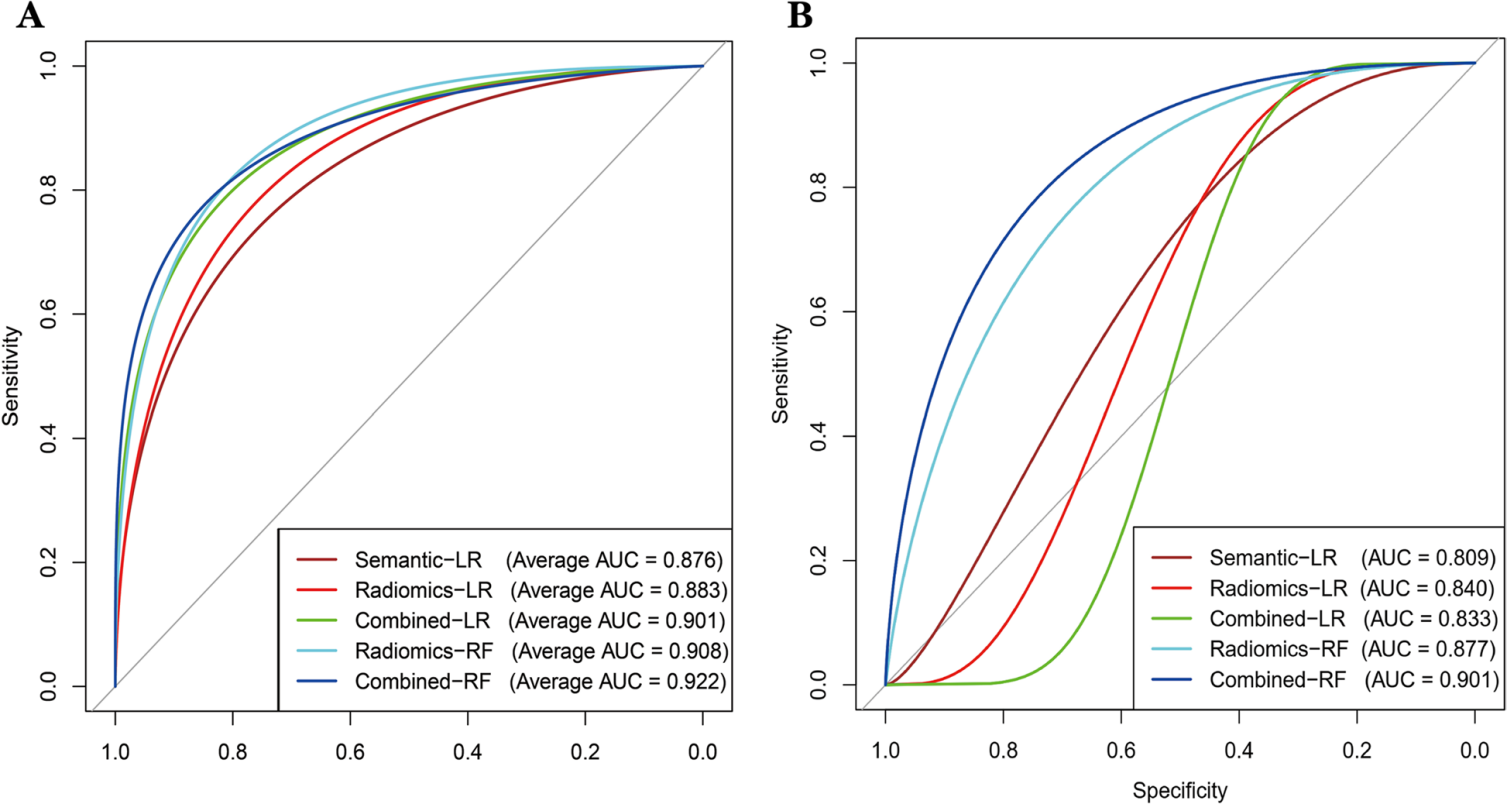
The ROC curves of all models for (**A**) five-fold cross-validation and (**B**) hold-out test. The average AUCs for the five-fold cross-validation represent the mean AUC values calculated across the five individual folds. ROC, receiver operating characteristic; LR, logistic regression; RF, random forest; Combined, semantic-radiomics combined; AUC, the area under the curve

The LR and RF algorithms were used to develop the semantic, radiomics, and semantic-radiomics combined models. The models validated using fivefold cross-validation demonstrated satisfactory performance. Both the semantic and radiomics LR models achieved high AUC values, with averages of 0.876 (range: 0.781–0.961) and 0.883 (range: 0.798–0.966) and the best performance, respectively. Additionally, they exhibited average accuracies of 0.878 (range: 0.839–0.952) and 0.901 (range: 0.825–0.968), respectively. The semantic-radiomics combined LR model outperformed the individual LR models, with an average AUC and accuracy of 0.901 (range: 0.862–0.927) and 0.907 (range: 0.871–0.952), respectively. When applying the RF algorithm, the radiomics model achieved an even higher average AUC value of 0.908 (range: 0.837–0.966), with an average accuracy of 0.894 (range: 0.825–0.952). The semantic-radiomics RF model had the highest average AUC of 0.922 (range: 0.872–0.982), exhibiting an average accuracy of 0.898 (range: 0.855–0.968) (Table 4). The hold-out method showed similar results; the radiomics RF model (AUC, 0.877; 95% CI, 0.861–0.970) had a greater AUC value than the semantic LR model (AUC, 0.809; 95% CI, 0.772–0.973), radiomics LR model (AUC, 0.840; 95% CI, 0.750–0.954), and semantic-radiomics combined LR model (AUC, 0.833; 95% CI, 0.725–0.957). The semantic-radiomics combined RF model exhibited the highest AUC value of 0.901 (95% CI, 0.886–0.968), with an accuracy of 0.947 (95% CI, 0.842–0.983) (Table 5). The AUC values of the RF models were generally higher than those of the LR models (Fig. 5, Figure E1).

**Table 4:** Accuracy of the models on the five-fold cross-validation test

Models	Average	Fold-1	Fold-2	Fold-3	Fold-4	Fold-5
Semantic-LR	0.878	0.839	0.873	0.889	0.839	0.952
Radiomics-LR	0.901	0.968	0.952	0.905	0.825	0.855
Combined^a^-LR	0.907	0.871	0.873	0.952	0.903	0.937
Radiomics-RF	0.894	0.935	0.952	0.825	0.871	0.889
Combined-RF	0.898	0.967	0.889	0.855	0.857	0.921

All statistics were validated in the test set of one institution

*AUC* the area under the curve, *CI* confidence interval, *LR* logistic regression, *RF* random forest

^a^semantic-radiomics combined

**Table 5:** Performance of the models on the hold-out test

Models	AUC (95% CI)	Accuracy (95% CI)
Semantic-LR	0.809 (0.772–0.973)	0.912 (0.798–0.947)
Radiomics-LR	0.840 (0.750–0.954)	0.947 (0.825–0.983)
Combined^a^-LR	0.833 (0.725–0.957)	0.965 (0.877–0.983)
Radiomics-RF	0.877 (0.861–0.970)	0.930 (0.814–0.965)
Combined-RF	0.901 (0.886–0.968)	0.947 (0.842–0.983)

All statistics were validated in the test set of one institution

*AUC* the area under the curve, *CI* confidence interval, *LR* logistic regression, *RF* random forest

^a^semantic-radiomics combined

## Discussion

In this study, we constructed LR and RF models using radiomic and/or descriptive semantic features to diagnose cervical LNM in patients with NSCLC. The findings are encouraging and expected to yield novel ideas for the noninvasive and precise diagnosis of cervical LNM. Three major findings were observed. First, ultrasound descriptive semantic features, including the long diameter, shape, and corticomedullary boundary, were independent risk factors for cervical LNM in NSCLC. Second, the radiomics model exhibited superior performance relative to the semantic model in diagnosing cervical LNM, and combining radiomic and descriptive semantic features yielded better performance than the single models. Third, the RF algorithm outperformed the LR algorithm in the development of models diagnosing cervical LNM in NSCLC.

Ultrasound is a noninvasive method for diagnosing cervical LN status in patients with NSCLC [8]. Ultrasound descriptive semantic features observed by radiologists are widely used in current clinical practice to identify the status of cervical LNs. Normal cervical LNs exhibit a flat or kidney-bean-shaped morphology and a hilum rich in fat [20]. In contrast, metastatic LNs display a rounded shape and an indistinct boundary on ultrasound imaging [21]. Our research found that a larger long diameter (average, 22.9 mm), irregular shape, and unclear corticomedullary boundary were independent risk factors for cervical LNM in NSCLC. In another study, the long diameter was identified as a critical risk factor for cervical LNM in nasopharyngeal carcinoma, with an average of 23 mm [22]. These results suggest that a 23 mm long diameter may be a suitable cutoff value for metastatic cervical LNs, but further research with a larger sample size is needed to validate this finding. The short diameter, perpendicular to the long diameter, remains controversial as a marker identifying cervical LNM because it varies depending on location and patient sex [23]. In the current study, multivariate analysis showed that it was not significantly associated with the cervical LNM in NSCLC. Defined as the ratio between the short and long diameter (SD/LD) of the node, the shape index clinically indicates malignant LNs when it is greater than 0.5, particularly metastatic LNs [21]. The shape index in this study was visually evaluated by observing the SD/LD and boundary of the cervical LNs. Our results provide evidence in support of the idea that the shape index is applicable in the identification of cervical LNM in NSCLC. In addition, an unclear corticomedullary boundary caused by uneven thickening suggests LNM, and our study demonstrated its association with cervical LNM in NSCLC. Nonetheless, relying solely on a single ultrasound descriptive semantic feature may prove inadequate in differentiating between metastatic and nonmetastatic cervical LNs [23]. Integrating crucial descriptive semantic features might provide more accurate differentiation. Therefore, we constructed a semantic model using features including long diameter, shape, and corticomedullary boundary, which had been selected through multivariate analysis. Our results demonstrated that the semantic model performed well in distinguishing between patients with NSCLC who had or did not have cervical LNM. Nevertheless, it should be noted that these descriptive semantic features are subjective and rely on the clinical expertise of the radiologists.

Radiomics can be used to extract quantitative features imperceptible to the naked eye from medical images, reflecting physiological, pathological, and genetic information in tumors [24, 25]. Although the use of radiomics in ultrasound is less common than in magnetic resonance imaging and computed tomography, an increasing number of studies have demonstrated the considerable potential of ultrasound-based radiomics for disease diagnosis and treatment [26, 27]. Using ultrasound-based radiomics, Zheng and her colleagues [27] reported a model that could predict the metastatic extent of the axillary lymph node in early-stage breast cancer. Wen and his colleagues [26] found that the model based on radiomic features outperformed the clinical model using independent clinical risk factors in predicting central cervical LNM in papillary thyroid carcinoma. Despite promising results in diagnosing cervical LN diseases, no studies have investigated the use of ultrasound radiomics for diagnosing cervical LNM in NSCLC until now. The current study represents the first project to employ ultrasound radiomics to diagnose cervical LNM in NSCLC, providing a novel noninvasive approach for clinical diagnosis. Consistent with previous studies, the fivefold cross-validated average AUC demonstrated the superiority of the radiomics model over the semantic model in diagnosing cervical LNM in NSCLC. Our results suggest that ultrasound radiomic features contain valuable information for diagnosing cervical LNM in patients with NSCLC.

Although our study demonstrated the excellent performance of the radiomics model, the clinical utility of descriptive semantic features should not be ignored. Combining the two types of features was shown to improve the performance of the individual models in this study. Min and his colleagues [22] presented a model that integrated radiomic and descriptive semantic features, achieving better performance than individual models in discriminating between benign and metastatic cervical LNs in patients with nasopharyngeal carcinoma. Consistent with their findings, our study found that the semantic-radiomics combined model performed better than individual models. These results highlight the added value of a combined approach in leveraging diverse information sources to achieve more accurate and robust classification. However, Min and his colleagues employed only the conventional LR algorithm when constructing their model, and applying other machine learning algorithms may improve its performance.

Numerous studies have highlighted the strong capacity of machine learning algorithms to develop prediction and classification models [28, 29]. In our previous study, we developed RF models based on radiomic features to classify thymomas and thymic carcinomas and distinguish early and advanced TNM stages of thymic epithelial tumors, with satisfactory performance [25]. Thus, we also employed the RF algorithm to construct models in the current study. The RF algorithm generates multiple decision trees and outputs the classification representing the predominant mode of the constituent trees during training. The ability of the RF algorithm to capture nonlinear interactions in the data makes it helpful in addressing complex and nonlinear relationships between variables. In contrast, the LR performs worse than the RF in analyzing nonlinear relationships and is easily affected by extreme values [30]. Our result is consistent with previous studies, as the RF model outperformed the LR model, emphasizing the robustness and effectiveness of the RF algorithm in the context of complex radiomic features. Moreover, the MDA was utilized to evaluate the performance of each feature, enabling researchers to focus on those features with a more substantial impact on the overall performance of the model. The original_shape2D_Elongatios was a significant modeling feature with the highest MDA value. Calculated as the ratio of the maximum length to the minimum length in the ROI shape, it underscores the critical role of the shape index in diagnosing cervical LNM in NSCLC. Additionally, 9 out of 15 features included in the RF model were wavelet features, accounting for the majority of the modeling features. This finding aligns with prior research incorporating wavelet features into radiomics models [31, 32]. The possible reason is that wavelet features may reflect spatial heterogeneity at multiple scales within tumor regions, but further research is necessary to investigate their correlation with pathological information.

Our study has some limitations. First, this is a retrospective study, which may have resulted in selection bias. Although three institutions were included in this study, we did not divide the external dataset in the main text accordingly due to the limited sample size. The significance of the differences between models was not tested because it tends to be not statistically significant with a small sample size. Therefore, a prospective study with a large sample size would be necessary to generalize our findings. Second, it is important to note that most cervical LNs that underwent ultrasound and ultrasound-guided biopsy examinations were suspected of metastasis based on palpable enlargement in this study. This led to a higher rate of cervical LNM in the sample, which is unusual and not representative for general NSCLC cohorts. In addition, only 37 of 313 patients were negative cases, which may have influenced the robustness of the models. To mitigate this issue, increasing the number of negative cases through targeted recruitment or data augmentation techniques may be considered. Third, the patients from the three institutions were examined with different ultrasound devices, which may affect the reproducibility and reliability of the radiomic features. Unified and standardized acquisition and reconstruction parameters may help mitigate this problem. Fourth, we used conventional ultrasound only, so we could not evaluate blood flow and other parameters. Contrast-enhanced ultrasound can display tumor vascularization, while shear wave elastography can assess tissue hardness. Thus, incorporating multimodal ultrasound radiomics analysis may improve the final performance when differentiating between cervical LNM-positive and cervical LNM-negative groups. In the future, prospective studies with larger sample sizes are needed to improve the performance of the models for diagnosing cervical LNM in patients with NSCLC.

## Conclusions

In conclusion, ultrasound-based radiomics with or without descriptive semantic features has the potential to be used to diagnose cervical LNM in NSCLC patients accurately. The use of the RF algorithm can enhance the performance of the radiomics models. These findings indicate that the models may potentially reduce invasive diagnostic procedures and aid in selecting appropriate treatment strategies, thereby improving patient management for those with NSCLC.

### Supplementary Information

**Supplementary Material 1.**

## Data Availability

The datasets used or analyzed during the current study are available from the corresponding author on reasonable request. Requests to access these datasets could be directed to 22tiandong@wchscu.cn.

## Abbreviations

NSCLC: Non-small cell lung cancer
LN: Lymph node
LNM: Lymph node metastasis
LR: Logistic regression
RF: Random forests
ICC: Interclass correlation coefficient
ROI: Region of interest
LASSO: Least absolute shrinkage and selection operator
MDA: Mean decrease accuracy
