# Supplementary material for: Ultrasound-based radiomics machine learning models for diagnosing cervical lymph node metastasis in patients with non-small cell lung cancer: a multicentre study
Source: BMC Cancer. 2024 Apr 27;24:536. doi: 10.1186/s12885-024-12306-6 (PMC11055367; doi:10.1186/s12885-024-12306-6)
Supplement: Supplementary file 1 — Supplementary Material 1. [file 12885_2024_12306_MOESM1_ESM.docx]

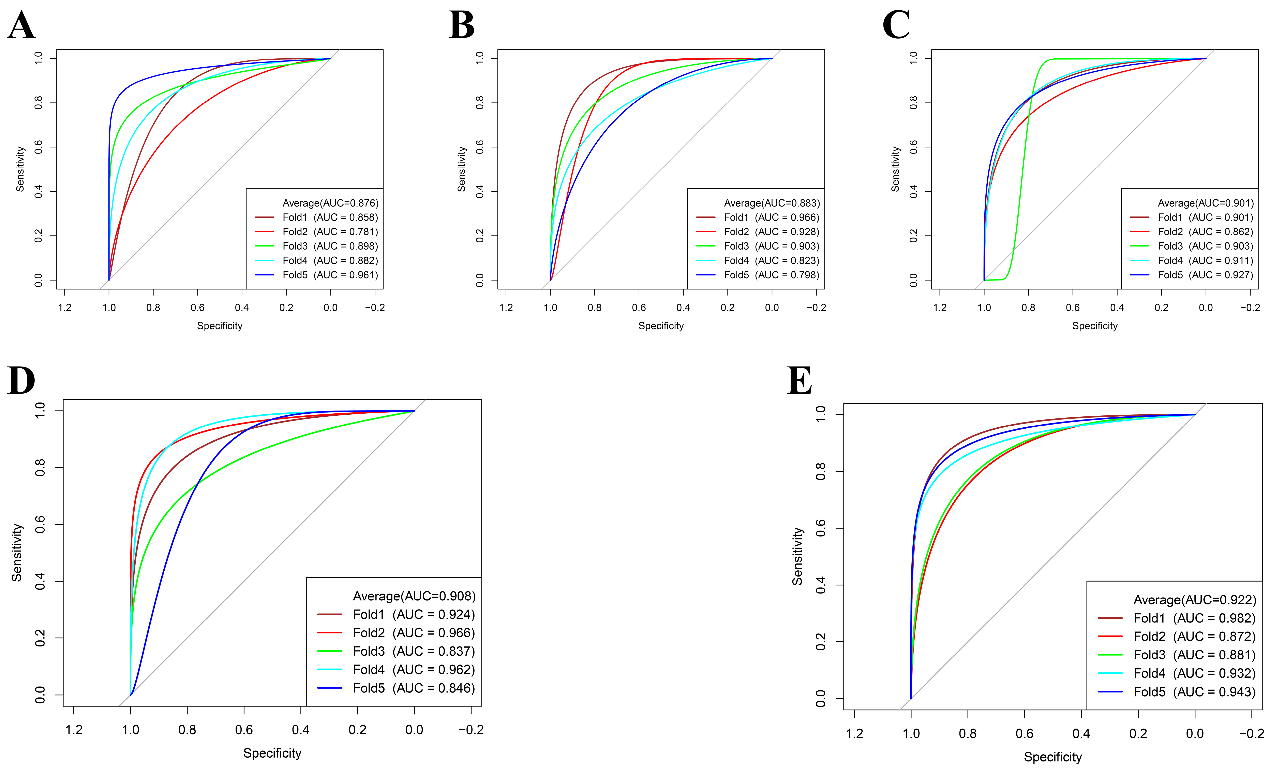


**Figure E1**. The ROC curves of all models. ROC, receiver operating characteristic. **A** the semantic logistic regression (LR) model; **B** the radiomics LR model; **C** the semantic-radiomics combined LR model; **D** the radiomics random forests (RF) model; **E** the semantic-radiomics combined RF model.
